# Advancing stroke therapy: the potential of MOF-based nanozymes in biomedical applications

**DOI:** 10.3389/fbioe.2024.1363227

**Published:** 2024-05-09

**Authors:** Meirong Chen, Yang Qin, Yongmei Peng, Ruyu Mai, Huanyao Teng, Zhongquan Qi, Jingxin Mo

**Affiliations:** ^1^ The Guangxi Clinical Research Center for Neurological Diseases, The Affiliated Hospital of Guilin Medical University, Guilin, China; ^2^ Medical College of Guangxi University, Nanning, China; ^3^ Department of Graduate and Postgraduate Education Management, The Affiliated Hospital of Guilin Medical University, Guilin, China; ^4^ School of Clinical Medicine, Guilin Medical University, Guilin, China; ^5^ Lab of Neurology, The Affiliated Hospital of Guilin Medical University, Guilin, China

**Keywords:** metal-organic frameworks, nanozymes, ischemic stroke treatment, reactive oxygen species, biomedical applications

## Abstract

In this study, we explored the growing use of metal-organic framework (MOF)-based Nanozymes in biomedical research, with a specific emphasis on their applications in stroke therapy. We have discussed the complex nature of stroke pathophysiology, highlighting the crucial role of reactive oxygen species (ROS), and acknowledging the limitations of natural enzymes in addressing these challenges. We have also discussed the role of nanozymes, particularly those based on MOFs, their structural similarities to natural enzymes, and their potential to improve reactivity in various biomedical applications. The categorization of MOF nanozymes based on enzyme-mimicking activities is discussed, and their applications in stroke therapy are explored. We have reported the potential of MOF in treating stroke by regulating ROS levels, alleviation inflammation, and reducing neuron apoptosis. Additionally, we have addressed the challenges in developing efficient antioxidant nanozyme systems for stroke treatment. The review concludes with the promise of addressing these challenges and highlights the promising future of MOF nanozymes in diverse medical applications, particularly in the field of stroke treatment.

## Graphical Abstract

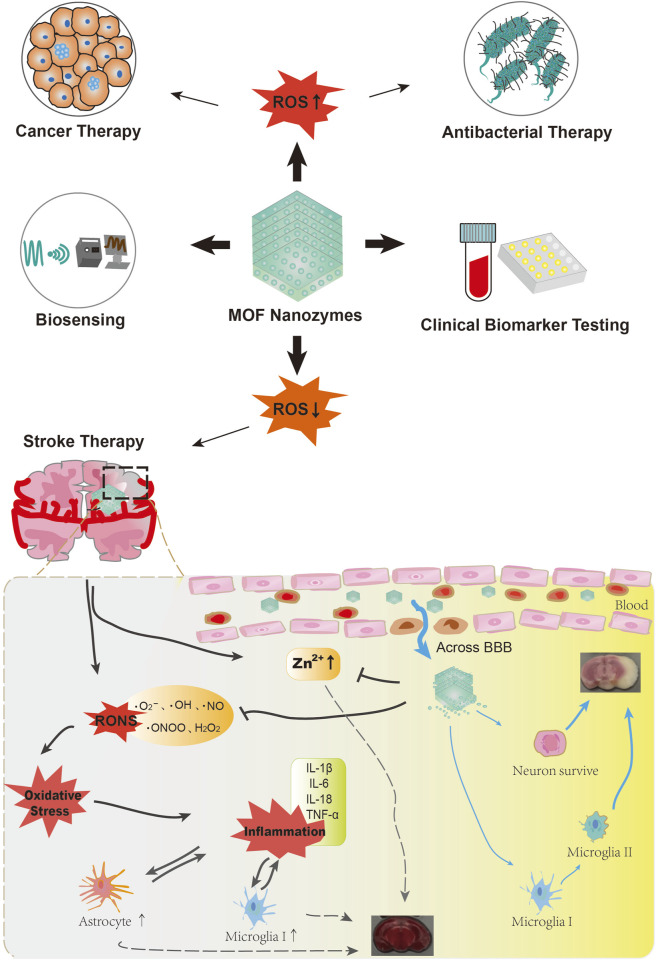

## 1 Introduction

Stroke, a cerebrovascular disease characterized by sudden neurological deficits, presents a significant global public health challenge due to its high incidence, disability, and mortality rates. Occurring predominantly in developing countries, approximately 87% of all stroke cases are ischemic strokes (IS) (Saini et al., 2021; Feigin et al., 2022). The pathophysiology of IS complex, involving excitotoxicity, mitochondrial dysfunction, autophagy dysregulation, oxidative stress, and neuroinflammation (Stonesifer et al., 2017; Tuo et al., 2022). Inflammation is a major factor in primary and secondary brain damage post-IS (Boltze and Perez-Pinzon, 2022). An essential aspect of this pathophysiology is the abnormal increase in the levels of reactive oxygen species (ROS), which is directly associated with oxidative stress and inflammatory responses. Therefore, alleviating ROS is crucial in IS treatment.

ROS, which naturally results from oxygen metabolism, play crucial roles in cell signaling and maintaining oxidative balance *in vivo* (Qi et al., 2022). An imbalance in the equilibrium between ROS production and elimination, whether due to antioxidant deficiency or ROS overproduction, can cause oxidative stress. This can contribute to several diseases such as aging, diabetes, cardiovascular, and inflammatory conditions, neurological disorders (including Parkinson’s disease and Alzheimer’s disease), and cancer (Chua et al., 2019; Talebi et al., 2022; Jomova et al., 2023).

Superoxide dismutase (SOD), catalase (CAT), and peroxidase (POD) are natural enzymes known for their efficacy in scavenging reactive oxygen species (ROS). These enzymes, composed of proteins or RNAs, catalyze specific reactions, reducing activation energy and thereby regulating metabolic, energy conversion, and disease processes (Wang et al., 2023). However, their applicability is limited to mild conditions, and they pose challenges in terms of separation, purification, cost, stability, and large-scale production (Chang et al., 2020). Nevertheless, there has been a shift in research toward the development of enzyme mimics, including catalytic cyclodextrins, polymers, supramolecules, porphyrins, and dendrimer macromolecules, aiming to emulate the functions of natural enzymes (Wulff, 2002; Wei and Wang, 2013).

In recent decades, the pursuit of mimicking the catalytic functions of natural enzymes has led to the emergence of artificial enzymes, which are designed to replicate the catalytic activities of their natural counterparts under laboratory conditions (Chen and Gridnev, 2020). Historically, artificial enzymes, including catalytic antibodies, peptide-based catalysts, and organic molecule-based catalysts, have offered promising avenues for research and applications (Zhang et al., 2019). However, challenges related to their stability, cost, and efficiency under physiological conditions have propelled the development of nanozymes.

Nanozymes, leveraging the advancements in nanotechnology, are engineered nanomaterials that exhibit enzyme-like activities (Singh, 2019; Villalba-Rodríguez et al., 2023). Offering advantages in terms of stability, affordability, and ease of preparation, nanozymes are positioned as ideal substitutes for natural enzymes. Over the last decade, nanozymes have been used in various fields, including biomedicine, food, and the environment (Ma et al., 2020; Shang et al., 2020; Curulli, 2021; Jiang et al., 2021; Khan et al., 2021; Ren et al., 2022; Wang et al., 2022). While most nanozymes exhibit oxidoreductase-like activities, some mimic SOD or CAT by scavenging ROS, whereas others function similarly to POD or oxidase (ODX) by generating ROS (Zhao et al., 2019; Liu et al., 2020). Despite the increasing variety of nanomaterials with enzyme-like activities, challenges persist in developing antioxidant nanozyme systems, such as achieving satisfactory catalytic activity, enabling multiple catalytic reactions, and ensuring good immunogenicity. Thus, antioxidant nanozymes with high activity, specificity, biosafety, and well-defined structures should be developed for therapeutic applications.

Among various nanozymes, Metal-Organic Frameworks (MOFs) based nanozymes stand out due to their unique structural features and tunable catalytic properties, offering new horizons in biomedical applications (Christodoulou et al., 2023). MOFs are ordered porous crystalline materials formed through the self-assembly of metal ions/clusters and multidentate ligands. They exhibit structural similarities to natural enzymes (Li et al., 2015; Abednatanzi et al., 2019). Their composition includes valence metals (serving as catalytic sites), organic ligands (acting as framework modulators), and pore structures (allowing mass transfer in catalytic reactions) (Lee and Telfer, 2023). MOFs have garnered increasing attention due to their high specific surface area, porosity, adjustable cavity structures, and biosafety. Their catalytic abilities are attributed to redox-active metal ions (such as Fe, Cu, Co, Ni, and Ce) and specialized organic ligands, which act as electronic mediators and mimic natural enzyme catalysis (Konavarapu et al., 2019; Wu et al., 2020; Li et al., 2021; Rojas-Buzo et al., 2021; Bohan et al., 2024). In this review, we have classified MOF-based nanozymes and provided a summary of their application in the medical field, with a specific focus on applications in stroke treatment.

## 2 Synthesis of metal-organic frameworks

**FIGURE 1:**
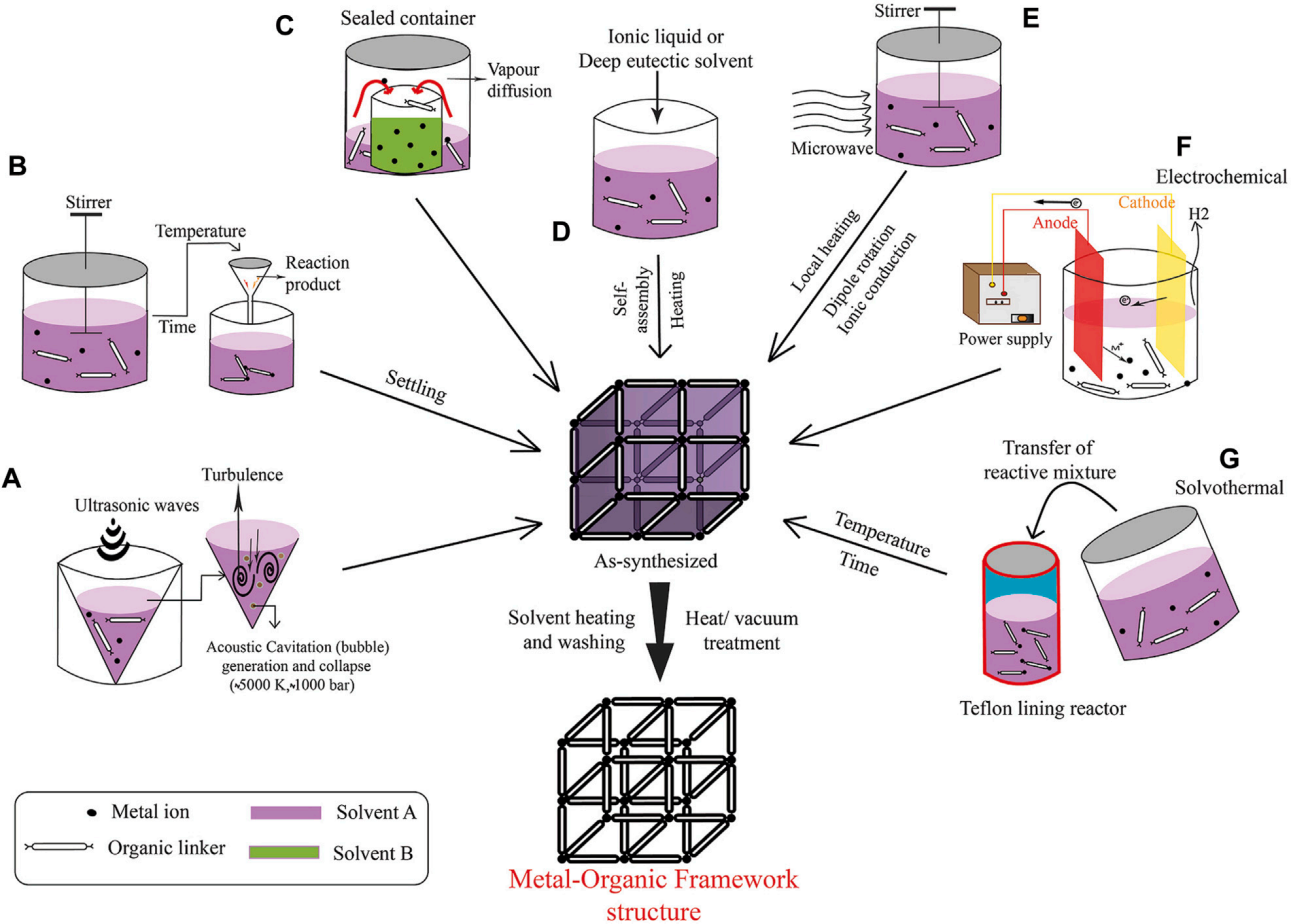
Overview of MOF Synthesis Techniques: **(A)** Sonochemical Approach, **(B)** Standard Solvothermal Method, **(C)** Vapor Diffusion Technique, **(D)** Iono-thermal Synthesis, **(E)** Microwave-Assisted Synthesis, **(F)** Electrochemical Assembly, and **(G)** Solvothermal Synthesis Process. (Referenced from (Sharanyakanth and Radhakrishnan, 2020)).

The synthesis of MOFs, as shown in Figure 1, can be achieved via various methods, each customized to attain specific structural and functional properties. One of the primary methods is solvothermal synthesis, wherein a metal salt is mixed with a multitopic organic linker in a high-boiling-point solvent such as N, N′–dimethylformamide (DMF), Diethyl formamide (DEF), or dimethyl sulfoxide, in a screw-top vial (Shi et al., 2004; Mohanty et al., 2010). The mixture is then heated, typically for 1–48 h. After completion of the reaction, the mixture is allowed to cool at room temperature. Subsequently, to remove impurities, the product is washed several times in succession with a deionized solution (such as water). The pure MOFs are obtained following centrifugation, washing with deionized solution, and vacuum drying.

Key parameters that can be changed during the process include reaction temperature, time, solvent, reagent concentration, pH, and the nature of the precursors. These factors can affect the topology, crystal size, and phase purity of the resulting MOF. Single crystals are readily available using this method, and single crystal x-ray diffraction can be used for structural characterization. Therefore, this method is highly selective for synthesizing MOF. This method can be used for the synthesis of porphyrin MOFs (PMOFs) and meso-tetra (4-carboxyphenyl) porphyrin (TCPP) MOF (Yan et al., 2023; Yu et al., 2024).

In instances where metal–ligand bonds exhibit exceptional strength, modulators are used to prevent the rapid precipitation of amorphous material. Modulators, such as benzoic acid, acetic acid, or hydrochloric acid, establish dynamic bonds with the metal precursor, competing with the linkers for metal coordination sites (Sugamata et al., 2020; Mao et al., 2022). They play a crucial role in the synthesis of Zr-MOFs, which contain robust Zr (IV)−O bonds. The selection of the modulator, its chemical composition, and concentration can significantly affect the defects, crystal size, habit, and topology of the MOF.

An alternative approach to traditional MOF synthesis involves the preformation of metal nodes or secondary building units (SBUs) (Zhou et al., 2018; Bour et al., 2020). This method includes the initial synthesis and isolation of metal cluster nodes, which are mixed with a tetratopic porphyrin-based linker and an acid modulator in DMF. Moreover, the preformation of metal clusters can optimize the phase purity and surface area of the MOF.

Other strategies for MOF synthesis include electrochemical, mechanochemical, sonochemical methods, and microwave-assisted synthesis (Hwang et al., 2005; Ni and Masel, 2006; James et al., 2012; Campagnol et al., 2014; Phang et al., 2014; Sharanyakanth and Radhakrishnan, 2020). To create MOF thin films, methods such as layer-by-layer deposition, liquid phase epitaxial growth, or seeded growth on a coated substrate are used. Moreover, post-synthetic methods, such as post-synthetic modification (PSM), solvent-assisted linker exchange (SALE), and transmetalation, enable the replacement of organic linkers or metal nodes in an existing MOF to create a new framework with the same topology.

Although the same reaction mixture (such as metal source, organic ligand, and solvent) is used in the formation of MOFs, the structures can differ due to differences in reaction time, particle size, yield, and morphology. Therefore, synthetic MOFs that are synthesized by different methods can vary. Different synthesis methods have their own advantages and disadvantages. Additionally, several MOF materials can be produced by various techniques that mix a large amount of available components and through variable process parameters.

## 3 MOF-based enzyme simulation system

MOFs, characterized by their unique porous structures and versatile functionalities, have emerged as promising tools for simulating enzymes. The majority of reported MOF nanozymes exhibit activities similar to oxidoreductases and resemble natural enzymes. For example, nanozymes mimicking the activities of SOD and CAT can scavenge ROS, protecting against oxidative stress. Conversely, those emulating POD and OXD can generate ROS, targeting harmful entities such as tumor cells and bacteria. This dual ability to both scavenges and generate ROS broadens the applicability of MOFs in biomedicine. Table 1 below classifies nanozymes based on their enzymatic activities and highlights their applications in the field of biomedicine.

**TABLE 1:** The main classification of MOF nanozymes.

Mimic enzyme	Classification	Application	Reaction principle	References
OXD	Ce-MOF	Detection of biothiols in serum samples and of dopamine in sweat samples	Colorimetric detection, colorimetric sensing	Xia et al. (2022), Xiong et al. (2015)
	Co-MOF	Detection of ultra-trace triazine endocrine disruptors and differentiation of aminophenol isomers	Colorimetric detection	Du et al. (2023), Ren et al. (2023)
	Cu-MOF	Screening for alpha-glucosidase inhibitors	Colorimetric sensing	Zhong et al. (2020)
	MnO-MOF	Cholesterol level determination	Catalytic oxidation	Xu et al. (2021)
	Co/2Fe-MOF	Sialic acid test	Oxidation reaction	Kıyıkçı et al. (2023)
POD	Fe-MOF	Alzheimer’s treatment	Bio-optical sensing	Miao et al. (2022)
	Cu-MOF	Optical biosensor detects C-reactive protein, anti-cancer	Colorimetric (fluorescence) detection, Fenton-like reaction	Hao et al. (2021), Ali and Omer (2022)
	Ni-MOF	Label-free fluorescence detection of hydrogen peroxide and glucose	Non-fluorescent labelling of hydrogen ions	Guo et al. (2022)
	Zr-MOF	Phosphorylated protein differentiation	Colorimetric sensing	Wang et al. (2020)
	Tb-MOF	Detection and degradation of estrogenic endocrine disruptors	Oxidative degradation	Wang and Chen (2020)
	Ni/Fe-MOF	Detection of hydrogen peroxide and glutathione	Colorimetric detection	Li et al. (2021)
	Au/Fe-MOF	Determination of prostate-specific antigen	Fenton-like reaction	Feng et al. (2020)
	Fe/Eu-MOF	Dual-mode alkaline phosphatase sensor	Accelerated fluorescent quenching	Shi et al. (2021)
	MOF-818	Detection of H_2_O_2_ and H_2_S levels released from living cells	Colorimetric and electrochemical dual-mode sensor	Yu et al. (2023)
Glutathione Peroxidase (GPx)	MIL-47(V)-NH2	Alleviate the inflammatory response effectively for both ear injury and colitis	Maintaining the reactive oxygen metabolic balance and protecting against injury by removing the excess H_2_O_2_	Wu et al. (2021)
CAT	Ce-MOF	Protection against iron overload damage in thalassemia and cancer	Elimination of ROS and iron overload, catalytic ATP depletion	Duan et al. (2023), Zhe et al. (2023)
	Mn-MOF	Enhancement of anti-tumor immunity and improvement of the immunosuppressive microenvironment; inflammatory bowel disease therapy	Promotion of ROS and iron death formation, thereby assisting in cancer inhibition and ROS-removal via sonodynamic therapy	Xu et al. (2021), Chen et al. (2022)
	Fe-MOF	Photodynamic therapy against tumors	Promoting ROS formation and assisting in photodynamic therapy for cancer inhibition	Liang et al. (2023)
	PCN-224-Pt	Cancer photodynamic therapy	Induction of H_2_O_2_ decomposition in tumors to generate ^1^O_2_, leveraging the cytotoxic potential of the produced ^1^O_2_ for cancer cell eradication	Zhang et al. (2018)
	Cu-MOF	Monitoring and management of bacterial-infected wounds	Decomposition of hydrogen peroxide	Mo et al. (2023)
SOD	Cu-MOF	Modelling superoxide anion sensing and removal of superoxide anion	Scavenging the oxygen catalytic activity	Guan et al. (2023)
	Ce-MOF	Protecting against iron overload damage in thalassemia	ROS-removal and iron-overload elimination	Duan et al. (2023)
Hydrolase	Ce-MOF	Glycopeptide analysis, prothrombin assay	Catalyzing self-cascading reactions	Pu et al. (2020), Yu et al. (2018)
Glucose oxidase (GOD)	TGZ@eM	Cancer-starvation therapy for colon cancer	Delivering GOD to tumor cells and degrading glucose to disrupt the tumor’s nutrient supply	Zhang et al. (2018)
Multi-enzyme system	Mn_3_ [Co(CN)_6_]_2_ MOF	Antitumor	Exert POD-like and OXD-like activities, catalyze the generation of O_2_ from endogenous H_2_O_2_, and facilitate the conversion of O_2_ into cytotoxic ROS	Wang et al. (2020)
	PyroFPSH	Photodynamic therapy for cancer	Overcome apoptosis resistance, reduce endogenous glutathione levels, and continuously generate ROS, due to remarkable multienzyme-like activities (GPx/CAT mimicry)	Lv et al. (2023)
	Fe-MIL-88NH_2_	Biofluid management and bacterial infection treatment	Excellent POD and OXD mimicry activities (the generation of •OH and •O_2_^−^ radicals)	Li et al. (2021)
	GATC	Monitoring and Management of Bacteria-Infected Wounds	With triple-enzyme activities, including POD, CAT and GPx mimicry, producing more •OH to kill bacteria, decomposing H2O2 into O2 to alleviate hypoxia and avoiding the loss of •OH for bacterial death more easily	Mo et al. (2023)
	Fe (III)-BTC-type MOF	Cascade colorimetric determination of glucose	With POD-like and GOD-like activities, during GOD’s enzymatic oxidation, glucose consumption, and H_2_O_2_ production, leading to the oxidation of 3,3′,5,5′-tetramethylbenzidine (TMB) by POD mimic, thereby forming a blue-green product	Zhao et al. (2019)

## 4 Catalytic mechanism of MOF-Based nanozymes

MOFs offer a unique blend of metal ion reactivity, organic linker functionality, and structural porosity that mimics the efficiency and specificity of natural enzymes (Islamoglu et al., 2017). The catalytic activity of MOF-based nanozymes is attributed to their hybrid composition of metal nodes acting as catalytic centers and organic linkers that enhance substrate specificity and catalytic efficiency (Zhang et al., 2019). These nanozymes are capable of performing electron transfer reactions akin to natural oxidoreductase enzymes, such as catalases and superoxide dismutases, demonstrating their potential in scavenging reactive oxygen species and offering new pathways for biomedical applications (Niu et al., 2020). Recent advancements highlight the potential of MOFs to incorporate multienzyme systems, achieving synergistic activities that could address oxidative stress-related disorders effectively (Bai et al., 2024). This confluence of features underscores MOFs’ versatility in biomedical fields, paving the way for the development of advanced therapeutic agents with tailored catalytic properties.

## 5 The application of nano-enzymes using MOF for stroke treatment

Stroke is a severe acute cerebrovascular disease, which is broadly classified into hemorrhagic stroke and IS. IS characterized by cerebral infarction or arterial blockage, causing symptoms such as hemiplegia or impaired consciousness. It is the leading cause of morbidity, recurrence, death, and disability (Kamtchum-Tatuene and Jickling, 2019; Kapoor et al., 2019). The current FDA-approved treatment for IS includes a tissue plasminogen activator, which can effectively dissolve thrombi within a narrow therapeutic window of 4.5 h. However, only a few patients can benefit from this. Another treatment option includes interventional therapy, which is limited by stringent technical and patient condition requirements and can pose risks such as cerebral hemorrhage (Kim, 2019).

### 5.1 Scavenging ROS and evading oxidative stress

The ‘sensitivity of the brain toward ischemia and hypoxia arises from its dependence on oxygen and glucose supplies, lacking significant energy reserves. Cerebral ischemia can cause hypoxia, rapid energy depletion, and subsequent nerve cell depolarization and excitatory neurotransmitter (glutamate) release (Dirnagl et al., 1999). This can trigger cellular excitotoxicity, resulting in cell swelling. Intracellular Ca^2+^ overload activates many enzyme systems, causing membrane disruption and the production of substantial amounts of ROS and reactive nitrogen species (RNS), which can activate apoptosis, necrosis, and autophagy, finally determining infarct size (Li et al., 2023). Oxidative stress is a result of ROS/free radicals and antioxidant system imbalance, which can be countered by natural antioxidants. Therefore, drugs with potent ROS-scavenging abilities should be developed (Betteridge, 2000; Li et al., 2018; Feigin et al., 2022).

**FIGURE 2:**
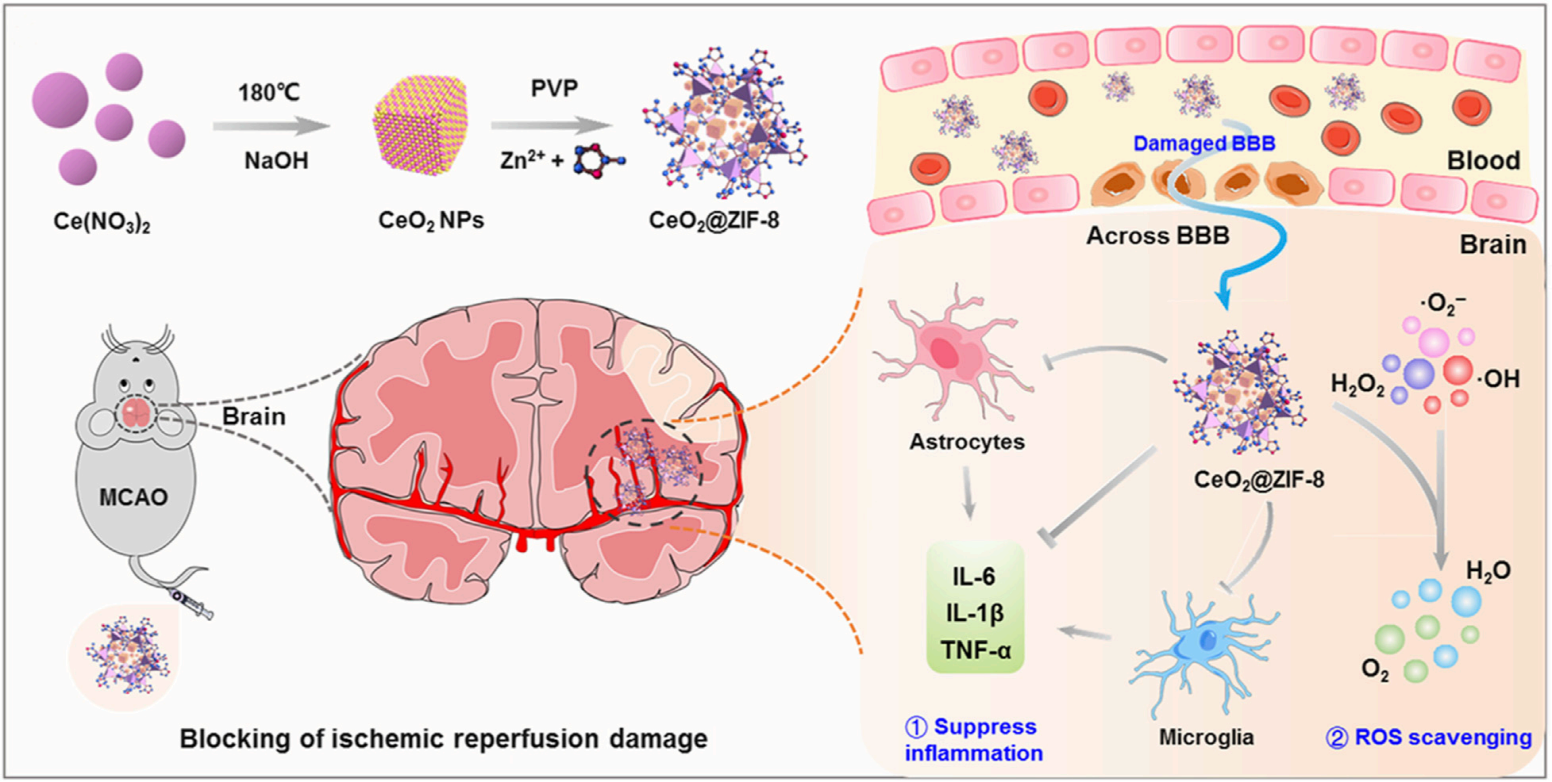
Schematic illustration for *in situ* synthetic approach of CeO2@ZIF-8 nanotherapeutics and its neuroprotective application mechanisms against reperfusion-induced injury in ischemic stroke. ref to (He et al., 2020).

Many nano-antioxidants, including ferric, manganese, magnetite, and cerium dioxide (CeO_2_) nanoparticles, have been constructed for stroke treatment (Kwon et al., 2018; Tao et al., 2019; Liu et al., 2021; Ma et al., 2023; Salatin et al., 2023; Wang et al., 2023). CeO_2_ NPs are particularly advantageous for their high antioxidant activity and recyclable ROS scavenging capability due to the fluorite lattice structure and the electron transfer between Ce^3+^ and Ce^4+^ (Bao et al., 2018; Li et al., 2022). ’A study by Kim revealed CeO_2_ NPs as effective free radical scavengers, which can prevent neuron damage from oxidative stress during IS (Kim et al., 2012). Nevertheless, challenges such as short vascular circulation time, interparticle aggregation, and direct catalytic reaction on active sites hinder their clinical application. Encapsulating CeO_2_ NPs with stable, biocompatible shells, such as zeolite imidazoline framework-8 (ZIF8), improves their properties and applicability (Zhu et al., 2021). As shown in Figure 2, He et al. innovatively synthesized ZIF-8-capped CeO_2_ NPs (CeO_2_@ZIF-8), which have improved catalytic and antioxidative activities. ZIF-8 mimics POD and can disintegrate or absorb H_2_O_2_, thereby exerting antioxidant activity. The CeO_2_ core containing the ZIF-8 layer is ideal for biological applications due to its controlled size, shape, and surface charge. The synergy between ZIF-8 decomposition and CeO_2_ release improves stroke treatment efficacy. Furthermore, CeO_2_@ZIF-8 NPs can effectively scavenge ROS (•OH, •O_2_^−^, and H_2_O_2_) and protect neuronal cells in MCAO model mice (He et al., 2020).

### 5.2 Integrated cascade nanozymes for stroke treatment

The development of cascade Nanozymes mimicking anti-ROS therapy is an effective strategy. ’Liu et al. established an integrated cascade nanozyme (Pt@PCN222-Mn), combining Mn-based MOF compounds (mimicking SOD) that can convert oxygen radicals into H_2_O_2_ and platinum nanoparticles that can (mimicking CAT) disproportionate H_2_O_2_ into water and oxygen. This cascade nanozyme exhibits excellent ROS scavenging ability in inflammatory bowel disease models (Liu et al., 2020).

Based on this aforementioned concept, Liu (Liu et al., 2020) designed an online electrochemical detection system using a rat cerebral ischemia model. This system used ZIF-67/Cu_0.76_Co_2.24_O_4_ nanospheres (ZIF-67/Cu_0.76_Co_2.24_O_4_ NSs) synthesized via alcohol heating with Cu(NO_3_)_2_. These nanospheres, possessing POD-like, SOD-like, GOD-like, and laccase-like activities, can effectively clear ROS and evade oxidative stress. They can catalyze 3,4-dihydroxyphenylacetic acid (DOPAC) into anthraquinones, allowing near real-time monitoring of DOPAC, a brain damage biomarker, in rat brain microdialysate.

In a previous study, Co-containing ZIF with cysteine-induced structural defects (ZIF-L-Co) was introduced, exhibiting improved catalytic activities of ascorbate oxidase and lyase. This helped in monitoring uric acid (UA) levels in the brain. The increased selectivity of the online electrochemical system for UA detection in rat brain microdialysate highlights the potential of this system for cerebral ischemia treatment and therapeutic effect assessment (Qu et al., 2019).

The use of MOF nano-enzymes in stroke treatment marks a promising shift in neuroprotective therapeutics, particularly in addressing IS. These innovative nano-enzymes, such as exhibited Pt@PCN222-Mn and ZIF-67/Cu_0.76_Co_2.24_O_4_ NSs, have remarkable efficacy in scavenging ROS and alleviating oxidative stress, pivotal factors in cerebral ischemia. Their ability to closely emulate natural antioxidant enzymes, along with enhanced stability and biocompatibility, highlights their potential to revolutionize stroke treatment. This advancement not only improves the effectiveness of presently used therapies but also paves the way for real-time monitoring and targeted intervention, allowing improved patient outcomes in cerebrovascular health.

### 5.3 Regulating reactive oxygen and nitrogen species and alleviating inflammatory response and apoptosis in stroke treatment

The overproduction of reactive oxygen and nitrogen species (RONS) in IS, such as •NO and •ONOO, can exacerbate brain damage. Excessive •NO can interact with •O_2_^−^, forming •ONOO and •OH and causing toxic effects (Ebrahimkhani et al., 2014; Yang et al., 2022). The inflammatory cascade is promptly triggered after vascular occlusion, which involves injury-associated molecular patterns and cytokines that can activate pattern recognition receptors (PRRs) on microglia and astrocytes (Gross et al., 2011). PRRs can detect injury-associated molecular patterns via toll-like receptors and inflammasomes (X. Y. Xiong et al., 2016).

Microglia activation, occurring within hours of an ischemic event, can cause the release of cytokines such as IL-1β, IL-6, IL-18, TNF-α, and NO, further perpetuating the inflammatory response (Jayaraj et al., 2019). Activated astrocytes can contribute to this process via the production of pro-inflammatory and anti-inflammatory cytokines (Ronaldson and Davis, 2012). Excessive accumulation of RONS can deactivate endogenous antioxidant enzymes, causing oxidative damage, especially in the ischemic penumbra (Schaller and Graf, 2004).

**FIGURE 3:**
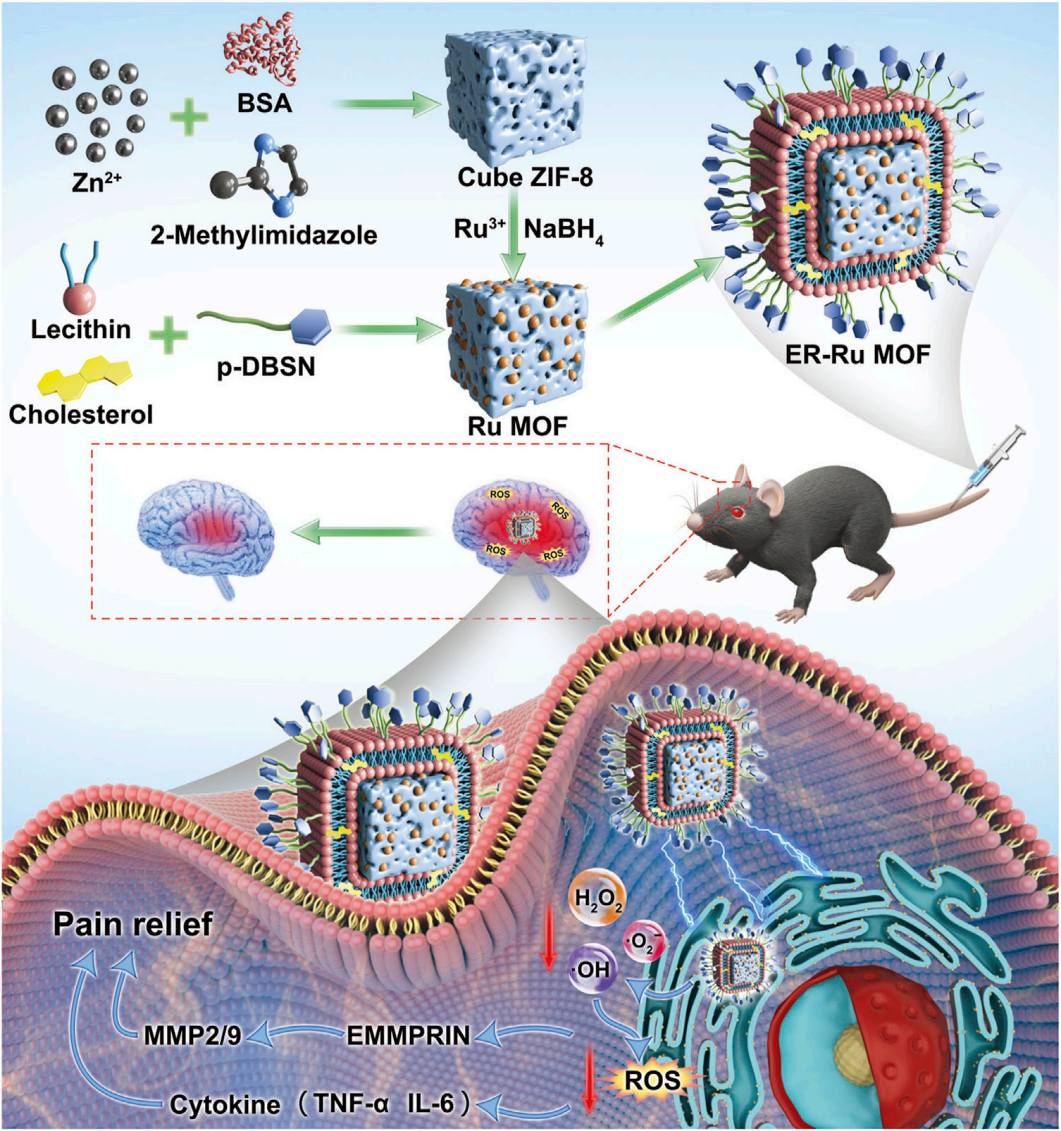
Schematic illustration of ER-Liposome encapsulated MOF tailored for therapeutic mechanisms on central post-stroke pain focused on oxidative stress regulation. (Referenced from (Bai et al., 2023)).

To address these limitations, Bai et al. (2023) constructed a ruthenium (Ru) MOF nanozyme encapsulated in an endoplasmic reticulum-targeted liposome (ER-Ru MOF), exhibiting SOD/CAT cascade catalytic activities, which can scavenge mitochondrial ROS and RNS. In a central post-stroke pain mouse model, ER-Ru MOF can significantly decrease the levels of pro-inflammatory cytokines and exert neuroprotective effects (Figure 3).

Prussian blue (PB) MOF Nanozymes are FDA-approved and known for their biosafety. They have also shown promising advantages in stroke treatment. PB Nanozymes can efficiently scavenge ROS due to their multiple enzyme-like activities (Estelrich and Busquets, 2021). Unlike iron-based nanoparticles, PB nanoparticles can inhibit •OH production, reducing toxicity (Zhao et al., 2018). Liu (J. Liu et al., 2023) demonstrated that PBzyme can inhibit macrophage activation, and neuronal cell apoptosis, and increase neurological recovery post-stroke by scavenging excess ROS.

**FIGURE 4:**
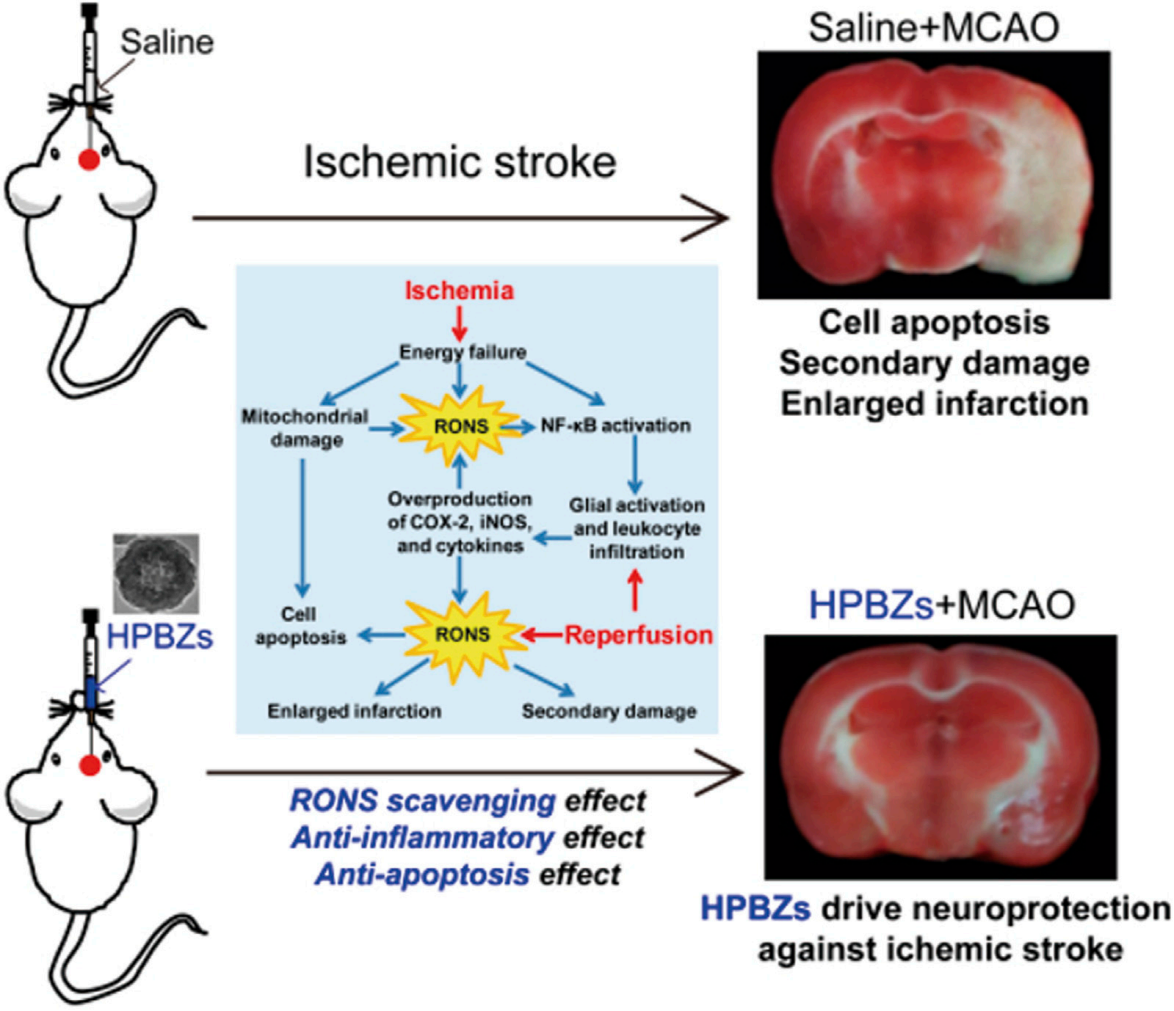
Schematic diagram of HPBZs driving neuroprotection against ischemia/reperfusion injury. (Reference (Zhang et al., 2019)).

Zhang et al. (2019) optimized the synthesis of hollow Prussian blue nanozymes (HPBZs) with multi-enzyme activity for RONS scavenging in a rat model of IS (Figure 4). HPBZs have a hollow structure and excellent redox ability, which can robustly scavenge RONS and convert them into harmless molecules. This can help in alleviating oxidative stress, apoptosis, and inflammation.

MOF nano-enzymes have emerged as potent therapeutic agents for stroke treatment, with a focus on scavenging ROS and RONS, as well as modulating inflammatory responses. Studies have reported the effectiveness of many MOF nano-enzymes in alleviating oxidative stress and inflammatory cytokines, protecting neuronal cells, and facilitating recovery in stroke models. The development of nano-enzymes, such as ER-Ru MOF and HPBZs, showcases the potential of MOFs in neuroprotection, emphasizing their role in advancing stroke therapy. This research lays the foundation for future innovations in the treatment of cerebral ischemia and other RONS-related diseases.

### 5.4 Regulating intracellular excess free Zn^2+^ and alleviating neuron apoptosis

Under normal physiological conditions, Zn^2+^ are protein-bound and play important roles in different biochemical functions, keeping free Zn^2+^ levels low (Frederickson et al., 2005). Nevertheless, during IS and reperfusion, an excess of protein-bound Zn^2+^ is released (Medvedeva et al., 2017). This increase in free Zn^2+^ can activate multiple pathways, such as glyceraldehyde-phosphate dehydrogenase (GAPDH) and glutathione reductase, thus triggering the transient receptor potential melatonin-associated 2 (TRPM2) pathway and resulting in neuronal cell injury and apoptosis (Mortadza et al., 2017). Addressing Zn^2+^ and ROS-associated cerebral ischemia-reperfusion injury (CIRI) is challenging because most studies focus on only one factor and cannot reduce the synergistic toxic effects on cells (Z. Guo et al., 2014).

Chai et al. developed a super-assembled MOF nanozyme system (2MI-P@MSN) to simultaneously detect, image, and chelate intracellular Zn^2+^, and scavenge ROS. This system, comprising polyethylene glycol (PEG)-modified mesoporous silica nanoparticles (MSN), can encapsulate 2-methylimidazole (2MI) and a Zn^2+^ probe (PZn), exhibiting a “turn-on” fluorescence signal for Zn^2+^ at 476 nm. 2MI chelated free Zn^2+^, assembling ZIF-8 intracellularly at the same site. Furthermore, 2MI-P@MSN is biocompatible and non-toxic and can effectively increase the survival rate of reperfusion-injured cells from 52% to 73%, while enabling selective quantitative Zn^2+^ detection in cells (Chai et al., 2021).

### 5.5 Preventing cerebral ischemia-reperfusion injury

The rapid restoration of blood perfusion is crucial in IS treatment; however, reperfusion can lead to secondary CIRI. Tian et al. synthesized a multi-enzyme cascade antioxidant system (Fe2NC@Se) via the encapsulation of a double iron atom nano-enzyme (Fe2NC) within a selenium-containing MOF (Se-MOF) shell layer. The Fe_2_NC@Se nano-enzymes, exhibiting SOD, CAT, and GPx-like activities, can effectively remove intracellular ROS and inhibit the ASK1/JNK apoptosis pathway. This resulted in a reduction of oxidative damage and neuronal apoptosis post-CIRI (Ruizhen Tian et al., 2022).

Cheng et al. established integrated nano-enzymes (INAzymes) by adding hemin and GOD in ZIF-8 nanostructures, improving catalytic activity and stability. ’The dual enzyme reaction of INAzyme facilitates glucose detection and monitoring of striatal glucose changes after cerebral ischemia/reperfusion in rats (Cheng et al., 2016).

### 5.6 Crossing the blood–brain barrier for treatment

**FIGURE 5:**
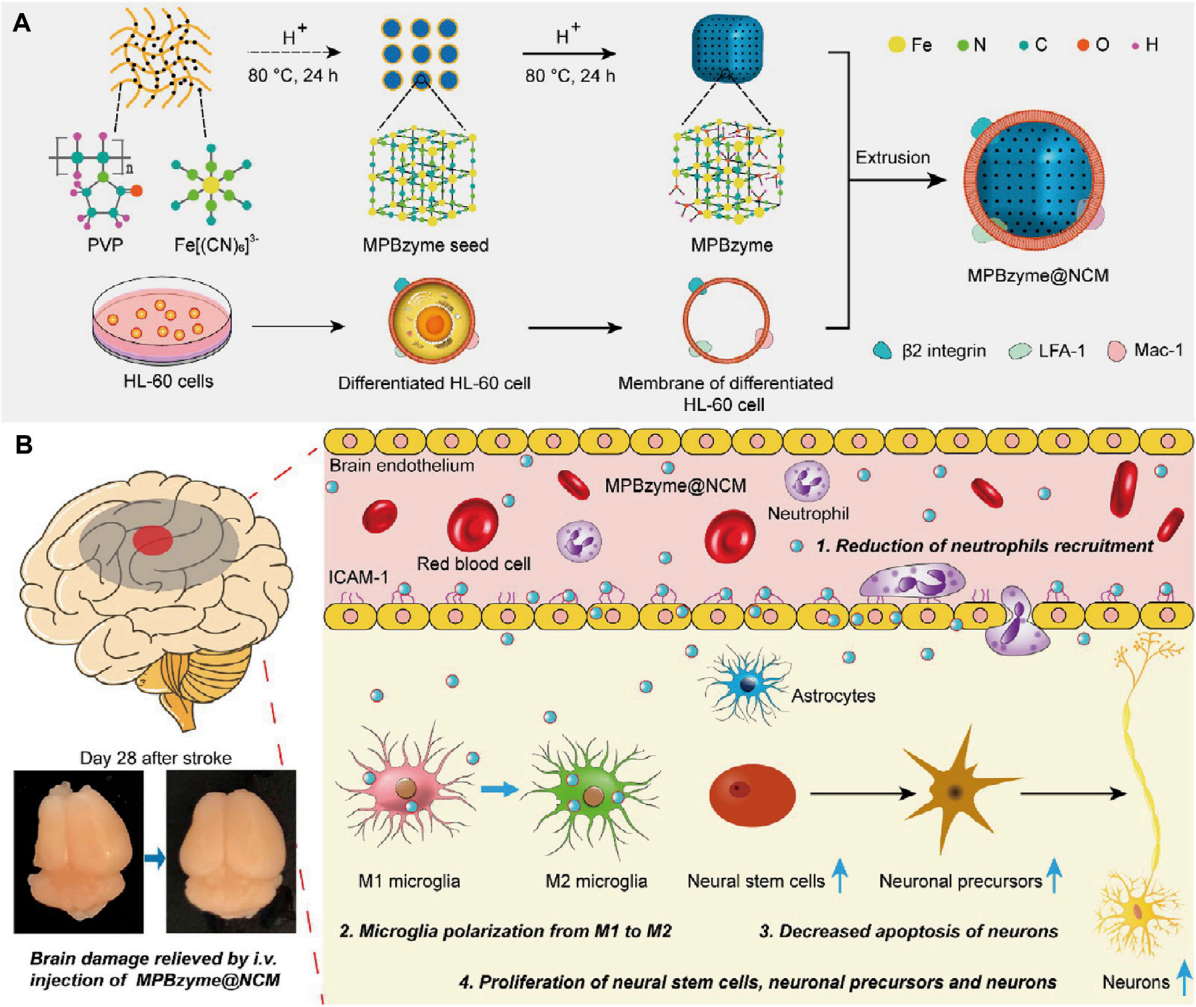
Schematic Representation of Neutrophil-Like Cell-Membrane (NCM)-Coated Nanozyme (MPBzyme@NCM) in the Treatment of Ischemic Stroke. **(A)** Scheme of the MPBzyme@NCM synthesis process. **(B)** Illustration of MPBzyme@NCM therapy for ischemic damage and long-term neurological functional recovery. Coating of NCM efficiently enhanced the penetration of nanozyme across brain microvascular endothelial cells. The MPBzyme@NCM elicited long-term therapeutic efficacy after stroke by reducing neutrophil recruitment, driving microglia polarization from M1 to M2, decreasing apoptosis of neurons, andincreasing the proliferation of neural stem cells, neuronal precursors, and neurons. (Referenced from (L. Feng et al., 2021)).

Penetrating the blood–brain barrier (BBB) is important in IS treatment. Although BBB can be disrupted by excessive ROS during ischemia-reperfusion in stroke, previous studies have shown that a damaged BBB persists only a few hours in the open state (X. Jiang et al., 2018; Sadeghian et al., 2019). Recent studies have reported that MOF Nanozymes possess potent anti-inflammatory and anti-oxidative properties; therefore, they can be used for the treatment of IS. Nevertheless, insufficient accumulation of MOF Nanozymes in the ischemic brain by non-invasive administration inhibits their application. Feng et al. constructed a neutrophil-like membrane-coated mesoporous Prussian blue MOF Nanozyme (MPBzyme@NCM), leveraging the natural association between inflamed brain microvascular endothelial cells and neutrophils post-stroke (Figure 5). This design improved BBB penetration and delivery of MOF Nanozymes to the ischemic brain. MPBzyme@NCM modulates microglia polarization, alleviates neuronal apoptosis, and promotes the proliferation of neural stem cells and precursors (L. Feng et al., 2021).

MOF nano-enzymes are a promising approach for stroke treatment. They can effectively alleviate oxidative stress and inflammatory responses while ensuring targeted delivery to affected brain regions. By scavenging ROS/RNS, chelating excess Zn^2+^, and crossing the BBB, these nanozymes provide a multifaceted strategy for the treatment of IS. Their novel designs and functionalities exhibit significant potential in addressing the complex pathophysiology of stroke, making way for advanced therapeutic options in neuroprotection and recovery. A summary of the advantages and mechanism of action of MOF Nanozymes in the treatment of stroke is shown in Table 2.

**TABLE 2:** The advantages of MOF nanozymes in stroke treatment and action mechanisms.

Classification	Advantages	Action mechanism	References
CeO2@ZIF-8	Improves drug stability, biocompatibility, and prolongs its circulation time within the bloodstream, thereby enhancing the catalytic and antioxidative activities	Encapsulates CeO_2_ NPs with stable, biocompatible shells, such as ZIF-8, to prolong the blood circulation time of CeO_2_, reduce the clearance rate, improve the penetration across the BBB, and enhances its accumulation in the brain tissues, ZIF-8, mimics POD, disintegrates or absorbs H_2_O_2_, thereby exerting antioxidant activity	He et al. (2020)
ZIF-67/Cu0.76Co2.24O4 NSs	Induces the formation of cascade-reaction systems with a high overall activity	Effectively clears ROS and abrogated oxidative stress with POD-like, SOD-like, GPx-like, and laccase-like activities	Liu et al. (2020)
	Enables near real-time monitoring of DOPAC, a brain-damage biomarker, in rat brain microdialysate	Catalyzes 3,4-dihydroxyphenylacetic acid (DOPAC) into anthraquinones	
ZIF-L-Co	Improves the selectivity of the online electrochemical system for UA detection in rat brain microdialysate	Enhances catalytic activities of ascorbate oxidase and lyase	Qu et al. (2019)
ER-Ru MOF	Facilitates better targeting and thus better drug concentration at the site of injury	Nanozyme encapsulated in an endoplasmic reticulum-targeted liposome ER-Ru MOF to thalamic hemorrhage pain, exhibiting SOD/CAT cascade catalytic activities to scavenge mitochondrial ROS and RNS	Bai et al. (2023)
(PB) MOF	Presents excellent biosafety	PB nanozyme with multienzyme activity (SOD-like and CAT-like) efficiently scavenge ROS, reduce toxicity of •OH and inhibit macrophage activation, neuronal cell apoptosis	Liu et al. (2023)
HPBZs	Increases brain tolerance of ischemic injury with minimal side effects	HPBZs, with their hollow structure and multienzyme activity, robustly scavenge RONS, converting them into harmless molecules and mitigating oxidative stress, apoptosis, and inflammation	Zhang et al. (2019)
2MI-P@MSN	Regulates intracellular excess free Zn^2+^ with excellent biocompatibility and non-cytotoxicity	2MI-P@MSN displays a “turn-on” fluorescence signal for Zn^2+^, chelate intracellular Zn^2+^	Chai et al. (2021)
		Besides, ZIF-8 with POD-like activity, effectually scavenge ROS	
Fe2NC@Se	Possesses multi-enzyme cascade antioxidant activity	Encapsulates a double iron atom nano-enzyme (Fe_2_NC) in a selenium-containing MOF (Se-MOF) shell layer, with SOD, CAT, and GPx-like activities, effectively eliminate intracellular ROS and inhibit the ASK1/JNK apoptosis pathway, thereby reducing oxidative damage and neuronal apoptosis post-CIRI	Ruizhen Tian et al. (2022)
INAzymes	Facilitates glucose detection and monitoring of striatal glucose changes after cerebral ischemia/reperfusion	Integrate nano-enzymes by embedding hemin and GOD in ZIF-8 nanostructures; the product of the first reaction can be used immediately as a substrate for the second reaction, which overcomes the problems of diffusion-limited kinetics and product instability and improves the catalytic activity and stability	Cheng et al. (2016)
MPBzyme@NCM	Enhances the BBB penetration and delivery of MOF Nanozymes to the ischemic brain	Realizes noninvasive active-targeting therapy for ischemic stroke using neutrophil-like cell-membrane-coated mesoporous MPBzyme@NCM, based on innate connection between inflamed brain microvascular endothelial cells and neutrophils after stroke	Feng et al. (2021)
		PB nanozyme with SOD/CAT-like activities efficiently scavenge ROS and decrease apoptosis of neurons	

## 6 Conclusions and future prospects of MOF-Based nanozymes

**FIGURE 6:**
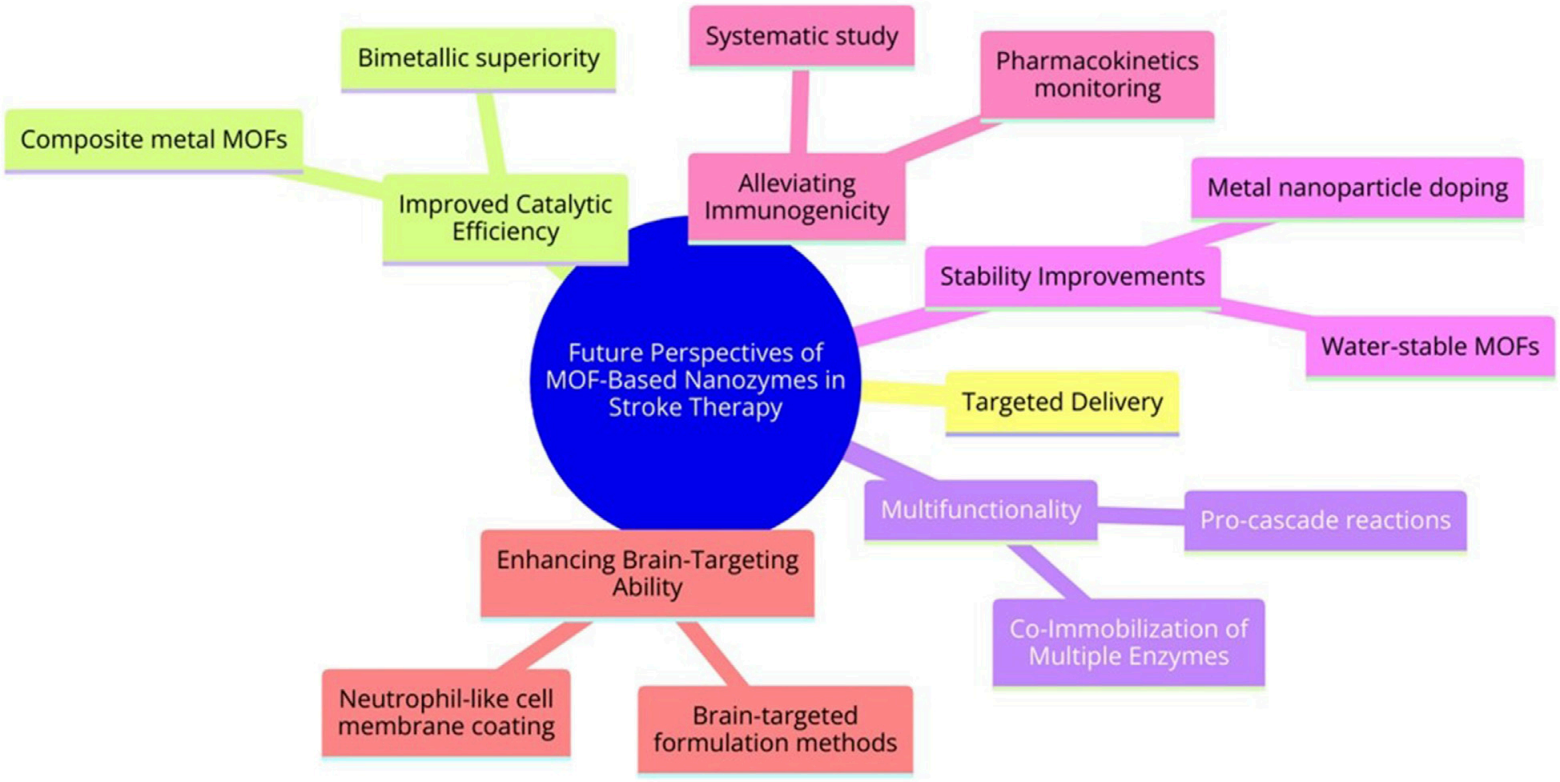
Future perspectives of MOF-Based nanozymes in stroke therapy.

MOF-based Nanozymes have made remarkable advancements in biomedical research, offering novel solutions to address limitations such as insufficient catalytic activity and low specificity. Nevertheless, optimizing their performance and expanding their applicability is still challenging. The key areas of focus are as follows (Figure 6):

a. **Improving Catalytic Activity:** MOF Nanozymes can catalyze a limited array of reactions, including specific redox and hydrolysis reactions, unlike the diverse biochemical reactions catalyzed by natural enzymes. Composite metal MOFs, especially bimetallic ones, have shown superior enzyme-like activity compared with monometallic MOFs. Adjusting the structural ratios of these composite metals can potentially optimize catalytic systems, thereby changing morphological structure, microstructure, and overall catalytic activity.
b. **Improving Stability:** Many MOFs synthesized in organic solutions show poor stability in aqueous environments and their backbone structure is prone to damage during catalysis. Therefore, more water-stable MOFs should be developed. Approaches such as reducing MOF material size or doping with metal nanoparticles (such as platinum) can improve reusability and broaden medical applications.
c. **Co-Immobilization of Multiple Enzymes:** The trend towards adding various catalytic agents, such as natural enzymes, Nanozymes, and metal nanoparticles, is garnering attention. Such multifunctional catalysts can allow pro-cascade reactions, reduce diffusion barriers, and maximize catalytic efficiency. Nevertheless, limitations, such as large particle sizes, inhomogeneous growth, and low utilization of catalytic sites in some MOF complexes, warrant further investigations.
d. **Alleviating Immunogenicity:** Although MOF nano-enzymes have made immense progress in stroke therapy due to their multi-enzyme catalytic activity, they have been studied only in small animals and are still far from clinical translation. Nevertheless, some limitations, such as immunogenicity, clinical toxicity, and poor pharmacokinetics, are still present. Therefore, the systematic study and real-time monitoring of the pharmacokinetics, biodegradation, and physiological parameters of MOF-derived Nanozymes after administration, and the assessment of long-term toxicity for further clinical translation should be the future focus.
e. **Improving Brain-Targeting Ability:** In the present study, only neutrophil-like cell membrane-coated MOF nano-enzyme was used to improve their brain-targeting ability. However, this method targets the site of inflammation and not the brain. If inflammation is present in other parts of the body, the drug enrichment can decrease at the ischemic inflammatory site of the brain. Therefore, using other brain-targeted formulation methods such as selecting suitable brain targets, formulation design and optimization, physical stimulation and penetration enhancement techniques, chemical modification and modification, and nasal administration can become effective measures to improve the brain-targeting properties of MOF nano-enzymes.

Despite these challenges, the ongoing studies on MOF-based Nanozymes are promising. These limitations will be surmounted with the advancements in the field, making the way for widespread development and application in stroke treatment. The role of MOF-based Nanozymes in these diverse fields indicates a promising future for their use in advanced medical and biotechnological applications.

## References

[B1] Abednatanzi S. Gohari Derakhshandeh P. Depauw H. Coudert F. X. Vrielinck H. Van Der Voort P. (2019). Mixed-metal metal-organic frameworks. Chem. Soc. Rev. 48 (9), 2535–2565. 10.1039/c8cs00337h 30989162

[B2] Ali G. K. Omer K. M. (2022). Ultrasensitive aptamer-functionalized Cu-MOF fluorescent nanozyme as an optical biosensor for detection of C-reactive protein. Anal. Biochem. 658, 114928. 10.1016/j.ab.2022.114928 36162448

[B3] Bai Q. Han Y. Khan S. Wu T. Yang Y. Wang Y. (2023). A novel endoplasmic reticulum-targeted metal-organic framework-confined ruthenium (Ru) nanozyme regulation of oxidative stress for central post-stroke pain. Adv. Healthc. Mater 13, e2302526. 10.1002/adhm.202302526 37823717

[B4] Bai Q. Han Y. Khan S. Wu T. Yang Y. Wang Y. (2024). A novel endoplasmic reticulum-targeted metal-organic framework-confined ruthenium (Ru) nanozyme regulation of oxidative stress for central post-stroke pain. Adv. Healthc. Mater 13 (2), e2302526. 10.1002/adhm.202302526 37823717

[B5] Bao Q. Hu P. Xu Y. Cheng T. Wei C. Pan L. (2018). Simultaneous blood-brain barrier crossing and protection for stroke treatment based on edaravone-loaded ceria nanoparticles. ACS Nano 12 (7), 6794–6805. 10.1021/acsnano.8b01994 29932327

[B6] Betteridge D. J. (2000). What is oxidative stress? Metabolism 49 (2 Suppl. 1), 3–8. 10.1016/s0026-0495(00)80077-3 10693912

[B7] Bohan A. Jin X. Wang M. Ma X. Wang Y. Zhang L. (2024). Uncoordinated amino groups of MIL-101 anchoring cobalt porphyrins for highly selective CO(2) electroreduction. J. Colloid Interface Sci. 654 (Pt B), 830–839. 10.1016/j.jcis.2023.10.089 37898067

[B8] Boltze J. Perez-Pinzon M. A. (2022). Focused update on stroke neuroimmunology: current progress in preclinical and clinical research and recent mechanistic insight. Stroke 53 (5), 1432–1437. 10.1161/strokeaha.122.039005 35467998

[B9] Bour J. R. Wright A. M. He X. Dincă M. (2020). Bioinspired chemistry at MOF secondary building units. Chem. Sci. 11 (7), 1728–1737. 10.1039/c9sc06418d 32180923 PMC7047978

[B10] Campagnol N. Souza E. R. De Vos D. E. Binnemans K. Fransaer J. (2014). Luminescent terbium-containing metal-organic framework films: new approaches for the electrochemical synthesis and application as detectors for explosives. Chem. Commun. (Camb) 50 (83), 12545–12547. 10.1039/c4cc05742b 25196133

[B11] Chai Q. Xie L. Gao M. Liu Y. Xu X. Huang X. (2021). Super-assembled silica nanoprobes for intracellular Zn(II) sensing and reperfusion injury treatment through *in situ* MOF crystallization. Analyst 146 (22), 6788–6797. 10.1039/d1an01475g 34671790

[B12] Chang C. C. Hsu T. L. Chen C. P. Chen C. Y. (2020). Enhancement of the peroxidase-like activity of iodine-capped gold nanoparticles for the colorimetric detection of biothiols. Biosens. (Basel) 10 (9), 113. 10.3390/bios10090113 PMC7558680 32882936

[B13] Chen G. Yu Y. Fu X. Wang G. Wang Z. Wu X. (2022). Microfluidic encapsulated manganese organic frameworks as enzyme mimetics for inflammatory bowel disease treatment. J. Colloid Interface Sci. 607 (Pt 2), 1382–1390. 10.1016/j.jcis.2021.09.016 34583043

[B14] Chen J. Gridnev I. D. (2020). Size is important: artificial catalyst mimics behavior of natural enzymes. iScience 23 (3), 100960. 10.1016/j.isci.2020.100960 32193144 PMC7076558

[B15] Cheng H. Zhang L. He J. Guo W. Zhou Z. Zhang X. (2016). Integrated nanozymes with nanoscale proximity for *in vivo* neurochemical monitoring in living brains. Anal. Chem. 88 (10), 5489–5497. 10.1021/acs.analchem.6b00975 27067749

[B16] Christodoulou I. Lyu P. Soares C. V. Patriarche G. Serre C. Maurin G. (2023). Nanoscale iron-based metal-organic frameworks: incorporation of functionalized drugs and degradation in biological media. Int. J. Mol. Sci. 24 (4), 3362. 10.3390/ijms24043362 36834775 PMC9965190

[B17] Chua L. Y. W. Chua B. L. Figiel A. Chong C. H. Wojdyło A. Szumny A. (2019). Characterisation of the convective hot-air drying and vacuum microwave drying of Cassia alata: antioxidant activity, essential oil volatile composition and quality studies. Molecules 24 (8), 1625. 10.3390/molecules24081625 31022967 PMC6515325

[B18] Curulli A. (2021). Electrochemical biosensors in food safety: challenges and perspectives. Molecules 26 (10), 2940. 10.3390/molecules26102940 34063344 PMC8156954

[B19] Dirnagl U. Iadecola C. Moskowitz M. A. (1999). Pathobiology of ischaemic stroke: an integrated view. Trends Neurosci. 22 (9), 391–397. 10.1016/s0166-2236(99)01401-0 10441299

[B20] Du B. Lu G. Zhang Z. Feng Y. Liu M. (2023). Glucose oxidase-like Co-MOF nanozyme-catalyzed self-powered sensor for sensitive detection of trace atrazine in complex environments. Anal. Chim. Acta 1280, 341817. 10.1016/j.aca.2023.341817 37858571

[B21] Duan Y. Liang L. Ye F. Zhao S. (2023). A Ce-MOF@polydopamine composite nanozyme as an efficient scavenger for reactive oxygen species and iron in thalassemia disease therapy. Nanoscale 15 (33), 13574–13582. 10.1039/d3nr01971c 37555269

[B22] Ebrahimkhani M. R. Daneshmand A. Mazumder A. Allocca M. Calvo J. A. Abolhassani N. (2014). Aag-initiated base excision repair promotes ischemia reperfusion injury in liver, brain, and kidney. Proc. Natl. Acad. Sci. U. S. A. 111 (45), E4878–E4886. 10.1073/pnas.1413582111 25349415 PMC4234618

[B23] Estelrich J. Busquets M. A. (2021). Prussian blue: a nanozyme with versatile catalytic properties. Int. J. Mol. Sci. 22 (11), 5993. 10.3390/ijms22115993 34206067 PMC8198601

[B24] Feigin V. L. Brainin M. Norrving B. Martins S. Sacco R. L. Hacke W. (2022). World stroke organization (WSO): global stroke fact sheet 2022. Int. J. Stroke 17 (1), 18–29. 10.1177/17474930211065917 34986727

[B25] Feng J. Wang H. Ma Z. (2020). Ultrasensitive amperometric immunosensor for the prostate specific antigen by exploiting a Fenton reaction induced by a metal-organic framework nanocomposite of type Au/Fe-MOF with peroxidase mimicking activity. Mikrochim. Acta 187 (1), 95. 10.1007/s00604-019-4075-4 31903507

[B26] Feng L. Dou C. Xia Y. Li B. Zhao M. Yu P. (2021). Neutrophil-like cell-membrane-coated nanozyme therapy for ischemic brain damage and long-term neurological functional recovery. ACS Nano 15 (2), 2263–2280. 10.1021/acsnano.0c07973 33426885

[B27] Frederickson C. J. Koh J. Y. Bush A. I. (2005). The neurobiology of zinc in health and disease. Nat. Rev. Neurosci. 6 (6), 449–462. 10.1038/nrn1671 15891778

[B28] Gross O. Thomas C. J. Guarda G. Tschopp J. (2011). The inflammasome: an integrated view. Immunol. Rev. 243 (1), 136–151. 10.1111/j.1600-065X.2011.01046.x 21884173

[B29] Guan L. Li B. Chen S. Ren G. Li K. Lin Y. (2023). Bioinspired Cu-based metal-organic framework mimicking SOD for superoxide anion sensing and scavenging. Talanta 265, 124860. 10.1016/j.talanta.2023.124860 37429254

[B30] Guo J. Liu Y. Mu Z. Wu S. Wang J. Yang Y. (2022). Label-free fluorescence detection of hydrogen peroxide and glucose based on the Ni-MOF nanozyme-induced self-ligand emission. Mikrochim. Acta 189 (6), 219. 10.1007/s00604-022-05313-6 35578119

[B31] Guo Z. Kim G. H. Yoon J. Shin I. (2014). Synthesis of a highly Zn(2+)-selective cyanine-based probe and its use for tracing endogenous zinc ions in cells and organisms. Nat. Protoc. 9 (6), 1245–1254. 10.1038/nprot.2014.086 24784821

[B32] Hao Y. N. Qu C. C. Shu Y. Wang J. H. Chen W. (2021). Construction of novel nanocomposites (Cu-MOF/GOD@HA) for chemodynamic therapy. Nanomater. (Basel) 11 (7), 1843. 10.3390/nano11071843 PMC8308241 34361229

[B33] He L. Huang G. Liu H. Sang C. Liu X. Chen T. (2020). Highly bioactive zeolitic imidazolate framework-8-capped nanotherapeutics for efficient reversal of reperfusion-induced injury in ischemic stroke. Sci. Adv. 6 (12), eaay9751. 10.1126/sciadv.aay9751 32206718 PMC7080448

[B34] Hwang Y. K. Chang J. S. Park S. E. Kim D. S. Kwon Y. U. Jhung S. H. (2005). Microwave fabrication of MFI zeolite crystals with a fibrous morphology and their applications. Angew. Chem. Int. Ed. Engl. 44 (4), 556–560. 10.1002/anie.200461403 15523677

[B35] Islamoglu T. Goswami S. Li Z. Howarth A. J. Farha O. K. Hupp J. T. (2017). Postsynthetic tuning of metal-organic frameworks for targeted applications. Acc. Chem. Res. 50 (4), 805–813. 10.1021/acs.accounts.6b00577 28177217

[B36] James S. L. Adams C. J. Bolm C. Braga D. Collier P. Friščić T. (2012). Mechanochemistry: opportunities for new and cleaner synthesis. Chem. Soc. Rev. 41 (1), 413–447. 10.1039/c1cs15171a 21892512

[B37] Jayaraj R. L. Azimullah S. Beiram R. Jalal F. Y. Rosenberg G. A. (2019). Neuroinflammation: friend and foe for ischemic stroke. J. Neuroinflammation 16 (1), 142. 10.1186/s12974-019-1516-2 31291966 PMC6617684

[B38] Jiang G. Wang Z. Zong S. Yang K. Zhu K. Cui Y. (2021). Peroxidase-like recyclable SERS probe for the detection and elimination of cationic dyes in pond water. J. Hazard Mater 408, 124426. 10.1016/j.jhazmat.2020.124426 33158654

[B39] Jiang X. Andjelkovic A. V. Zhu L. Yang T. Bennett M. V. L. Chen J. (2018). Blood-brain barrier dysfunction and recovery after ischemic stroke. Prog. Neurobiol. 163-164, 144–171. 10.1016/j.pneurobio.2017.10.001 28987927 PMC5886838

[B40] Jomova K. Raptova R. Alomar S. Y. Alwasel S. H. Nepovimova E. Kuca K. (2023). Reactive oxygen species, toxicity, oxidative stress, and antioxidants: chronic diseases and aging. Arch. Toxicol. 97 (10), 2499–2574. 10.1007/s00204-023-03562-9 37597078 PMC10475008

[B41] Kamtchum-Tatuene J. Jickling G. C. (2019). Blood biomarkers for stroke diagnosis and management. Neuromolecular Med. 21 (4), 344–368. 10.1007/s12017-019-08530-0 30830566 PMC6722038

[B42] Kapoor A. Lanctot K. L. Bayley M. Herrmann N. Murray B. J. Swartz R. H. (2019). Screening for post-stroke depression and cognitive impairment at baseline predicts long-term patient-centered outcomes after stroke. J. Geriatr. Psychiatry Neurol. 32 (1), 40–48. 10.1177/0891988718819859 30793663

[B43] Khan S. Sharifi M. Bloukh S. H. Edis Z. Siddique R. Falahati M. (2021). *In vivo* guiding inorganic nanozymes for biosensing and therapeutic potential in cancer, inflammation and microbial infections. Talanta 224, 121805. 10.1016/j.talanta.2020.121805 33379031

[B44] Kim C. K. Kim T. Choi I. Y. Soh M. Kim D. Kim Y. J. (2012). Ceria nanoparticles that can protect against ischemic stroke. Angew. Chem. Int. Ed. Engl. 51 (44), 11039–11043. 10.1002/anie.201203780 22968916

[B45] Kim J. S. (2019). tPA helpers in the treatment of acute ischemic stroke: are they ready for clinical use? J. Stroke 21 (2), 160–174. 10.5853/jos.2019.00584 31161761 PMC6549064

[B46] Kıyıkçı H. F. Avcı O. Tepeli Büyüksünetçi Y. Timur S. Anık Ü. (2023). Oxidase mimicking Co/2Fe MOF included biosensor for sialic acid detection. Talanta 254, 124166. 10.1016/j.talanta.2022.124166 36493566

[B47] Konavarapu S. K. Ghosh D. Dey A. Pradhan D. Biradha K. (2019). Isostructural Ni(II) metal-organic frameworks (MOFs) for efficient electrocatalysis of oxygen evolution reaction and for gas sorption properties. Chemistry 25 (47), 11141–11146. 10.1002/chem.201902274 31250943

[B48] Kwon H. J. Kim D. Seo K. Kim Y. G. Han S. I. Kang T. (2018). Ceria nanoparticle systems for selective scavenging of mitochondrial, intracellular, and extracellular reactive oxygen species in Parkinson’s disease. Angew. Chem. Int. Ed. Engl. 57 (30), 9408–9412. 10.1002/anie.201805052 29862623

[B49] Lee S. J. Telfer S. G. (2023). Multicomponent metal-organic frameworks. Angew. Chem. Int. Ed. Engl. 62 (44), e202306341. 10.1002/anie.202306341 37344359

[B50] Li A. Yang X. Chen J. (2021). A novel route to size-controlled MIL-53(Fe) metal-organic frameworks for combined chemodynamic therapy and chemotherapy for cancer. RSC Adv. 11 (18), 10540–10547. 10.1039/d0ra09915e 35423581 PMC8695691

[B51] Li J. S. Li S. L. Tang Y. J. Han M. Dai Z. H. Bao J. C. (2015). Nitrogen-doped Fe/Fe3C@graphitic layer/carbon nanotube hybrids derived from MOFs: efficient bifunctional electrocatalysts for ORR and OER. Chem. Commun. (Camb) 51 (13), 2710–2713. 10.1039/c4cc09062d 25575029

[B52] Li P. Stetler R. A. Leak R. K. Shi Y. Li Y. Yu W. (2018). Oxidative stress and DNA damage after cerebral ischemia: potential therapeutic targets to repair the genome and improve stroke recovery. Neuropharmacology 134 (Pt B), 208–217. 10.1016/j.neuropharm.2017.11.011 29128308 PMC5940593

[B53] Li Q. Wang Q. Li Y. Zhang X. Huang Y. (2021). 2D bimetallic Ni/Fe MOF nanosheet composites as a peroxidase-like nanozyme for colorimetric assay of multiple targets. Anal. Methods 13 (17), 2066–2074. 10.1039/d1ay00281c 33955987

[B54] Li W. Shao C. Li C. Zhou H. Yu L. Yang J. (2023). Metabolomics: a useful tool for ischemic stroke research. J. Pharm. Anal. 13 (9), 968–983. 10.1016/j.jpha.2023.05.015 37842657 PMC10568109

[B55] Li X. Han Z. Wang T. Ma C. Li H. Lei H. (2022). Cerium oxide nanoparticles with antioxidative neurorestoration for ischemic stroke. Biomaterials 291, 121904. 10.1016/j.biomaterials.2022.121904 36403323

[B56] Li Y. Wang D. Wen J. Yu P. Liu J. Li J. (2021). Chemically grafted nanozyme composite cryogels to enhance antibacterial and biocompatible performance for bioliquid regulation and adaptive bacteria trapping. ACS Nano 15 (12), 19672–19683. 10.1021/acsnano.1c06983 34878257

[B57] Liang Z. Li X. Chen X. Zhou J. Li Y. Peng J. (2023). Fe/MOF based platform for NIR laser induced efficient PDT/PTT of cancer. Front. Bioeng. Biotechnol. 11, 1156079. 10.3389/fbioe.2023.1156079 37064235 PMC10098195

[B58] Liu J. Sun J. Song Y. Wang M. Zhao P. Wang W. (2023). Prussian blue nanozyme treatment of ischemic brain injury via reducing oxidative stress inhibits inflammation, suppresses apoptosis, and promotes neurological recovery. ACS Chem. Neurosci. 10.1021/acschemneuro.3c00144 37038049

[B59] Liu J. Zhang W. Peng M. Ren G. Guan L. Li K. (2020). ZIF-67 as a template generating and tuning "raisin pudding"-type nanozymes with multiple enzyme-like activities: toward online electrochemical detection of 3,4-dihydroxyphenylacetic acid in living brains. ACS Appl. Mater Interfaces 12 (26), 29631–29640. 10.1021/acsami.0c05667 32476405

[B60] Liu Y. Cheng Y. Zhang H. Zhou M. Yu Y. Lin S. (2020). Integrated cascade nanozyme catalyzes *in vivo* ROS scavenging for anti-inflammatory therapy. Sci. Adv. 6 (29), eabb2695. 10.1126/sciadv.abb2695 32832640 PMC7439611

[B61] Liu Y. Wang X. Li X. Qiao S. Huang G. Hermann D. M. (2021). A Co-doped Fe(3)O(4) nanozyme shows enhanced reactive oxygen and nitrogen species scavenging activity and ameliorates the deleterious effects of ischemic stroke. ACS Appl. Mater Interfaces 13 (39), 46213–46224. 10.1021/acsami.1c06449 34546708

[B62] Lv L. Fu Z. You Q. Xiao W. Wang H. Wang C. (2023). Enhanced photodynamic therapy through multienzyme-like MOF for cancer treatment. Front. Bioeng. Biotechnol. 11, 1338257. 10.3389/fbioe.2023.1338257 38312507 PMC10834778

[B63] Ma X. Liu H. Wen S. Xie Q. Li L. Jin J. (2020). Ultra-sensitive SERS detection, rapid selective adsorption and degradation of cationic dyes on multifunctional magnetic metal-organic framework-based composite. Nanotechnology 31 (31), 315501. 10.1088/1361-6528/ab8a8f 32303010

[B64] Ma X. Zhang B. Ma N. Liu C. Miao Y. Liang X. (2023). Unveiling the mechanism of alleviating ischemia reperfusion injury via a layered double hydroxide-based nanozyme. ACS Appl. Mater Interfaces. 10.1021/acsami.2c19570 36914282

[B65] Mao W. Huang R. Xu H. Wang H. Huang Y. Huang S. (2022). Effects of acid modulators on the microwave-assisted synthesis of Cr/Sn metal-organic frameworks. Polym. (Basel) 14 (18), 3826. 10.3390/polym14183826 PMC9504004 36145971

[B66] Medvedeva Y. V. Ji S. G. Yin H. Z. Weiss J. H. (2017). Differential vulnerability of CA1 versus CA3 pyramidal neurons after ischemia: possible relationship to sources of Zn2+ accumulation and its entry into and prolonged effects on mitochondria. J. Neurosci. 37 (3), 726–737. 10.1523/jneurosci.3270-16.2016 28100752 PMC5242414

[B67] Miao Y. B. Zhong Q. Ren H. X. (2022). Engineering a thermostable biosensor based on biomimetic mineralization HRP@Fe-MOF for Alzheimer’s disease. Anal. Bioanal. Chem. 414 (29-30), 8331–8339. 10.1007/s00216-022-04367-y 36258085

[B68] Mo F. Zhong S. You T. Lu J. Sun D. (2023). Aptamer and DNAzyme-functionalized Cu-MOF hybrid nanozymes for the monitoring and management of bacteria-infected wounds. ACS Appl. Mater Interfaces. 10.1021/acsami.3c10682 37921634

[B69] Mohanty P. Linn N. M. Landskron K. (2010). Ultrafast sonochemical synthesis of methane and ethane bridged periodic mesoporous organosilicas. Langmuir 26 (2), 1147–1151. 10.1021/la902239m 19736985

[B70] Mortadza S. S. Sim J. A. Stacey M. Jiang L. H. (2017). Signalling mechanisms mediating Zn(2+)-induced TRPM2 channel activation and cell death in microglial cells. Sci. Rep. 7, 45032. 10.1038/srep45032 28322340 PMC5359577

[B71] Ni Z. Masel R. I. (2006). Rapid production of metal-organic frameworks via microwave-assisted solvothermal synthesis. J. Am. Chem. Soc. 128 (38), 12394–12395. 10.1021/ja0635231 16984171

[B72] Niu X. Li X. Lyu Z. Pan J. Ding S. Ruan X. (2020). Metal-organic framework based nanozymes: promising materials for biochemical analysis. Chem. Commun. (Camb) 56 (77), 11338–11353. 10.1039/d0cc04890a 32909017

[B73] Phang W. J. Lee W. R. Yoo K. Ryu D. W. Kim B. Hong C. S. (2014). pH-dependent proton conducting behavior in a metal-organic framework material. Angew. Chem. Int. Ed. Engl. 53 (32), 8383–8387. 10.1002/anie.201404164 24986637

[B74] Pu C. Zhao H. Hong Y. Zhan Q. Lan M. (2020). Facile preparation of hydrophilic mesoporous metal-organic framework via synergistic etching and surface functionalization for glycopeptides analysis. Anal. Chem. 92 (2), 1940–1947. 10.1021/acs.analchem.9b04236 31887020

[B75] Qi J. Y. Yang Y. K. Jiang C. Zhao Y. Wu Y. C. Han X. (2022). Exploring the mechanism of danshensu in the treatment of doxorubicin-induced cardiotoxicity based on network pharmacology and experimental evaluation. Front. Cardiovasc Med. 9, 827975. 10.3389/fcvm.2022.827975 35295262 PMC8918531

[B76] Qu S. Li Z. Jia Q. (2019). Detection of purine metabolite uric acid with picolinic-acid-functionalized metal-organic frameworks. ACS Appl. Mater Interfaces 11 (37), 34196–34202. 10.1021/acsami.9b07442 31456392

[B77] Ren M. Zhang Y. Yu L. Qu L. Li Z. Zhang L. (2023). A Co-based MOF as nanozyme with enhanced oxidase-like activity for highly sensitive and selective colorimetric differentiation of aminophenol isomers. Talanta 255, 124219. 10.1016/j.talanta.2022.124219 36580809

[B78] Ren X. Chen D. Wang Y. Li H. Zhang Y. Chen H. (2022). Nanozymes-recent development and biomedical applications. J. Nanobiotechnology 20 (1), 92. 10.1186/s12951-022-01295-y 35193573 PMC8864828

[B79] Rojas-Buzo S. Concepción P. Olloqui-Sariego J. L. Moliner M. Corma A. (2021). Metalloenzyme-inspired Ce-MOF catalyst for oxidative halogenation reactions. ACS Appl. Mater Interfaces 13 (26), 31021–31030. 10.1021/acsami.1c07496 34176269 PMC9131423

[B80] Ronaldson P. T. Davis T. P. (2012). Blood-brain barrier integrity and glial support: mechanisms that can be targeted for novel therapeutic approaches in stroke. Curr. Pharm. Des. 18 (25), 3624–3644. 10.2174/138161212802002625 22574987 PMC3918413

[B81] Ruizhen Tian H. M. Ye W. Li Y. Wang S. Zhang Z. Liu S. (2022). Se-containing MOF coated dual-Fe-atom nanozymes WithMulti-enzyme cascade activities protect against CerebralIschemic reperfusion injury. Adv. Funct. Mater. 32, 2204025. 10.1002/adfm.202204025

[B82] Sadeghian N. Shadman J. Moradi A. Ghasem Golmohammadi M. Panahpour H. (2019). Calcitriol protects the Blood-Brain Barrier integrity against ischemic stroke and reduces vasogenic brain edema via antioxidant and antiapoptotic actions in rats. Brain Res. Bull. 150, 281–289. 10.1016/j.brainresbull.2019.06.010 31220552

[B83] Saini V. Guada L. Yavagal D. R. (2021). Global epidemiology of stroke and access to acute ischemic stroke interventions. Neurology 97 (20 Suppl. 2), S6–s16. 10.1212/wnl.0000000000012781 34785599

[B84] Salatin S. Farhoudi M. Farjami A. Maleki Dizaj S. Sharifi S. Shahi S. (2023). Nanoparticle formulations of antioxidants for the management of oxidative stress in stroke: a review. Biomedicines 11 (11), 3010. 10.3390/biomedicines11113010 38002010 PMC10669285

[B85] Schaller B. Graf R. (2004). Cerebral ischemia and reperfusion: the pathophysiologic concept as a basis for clinical therapy. J. Cereb. Blood Flow. Metab. 24 (4), 351–371. 10.1097/00004647-200404000-00001 15087705

[B86] Shang Y. Liu F. Wang Y. Li N. Ding B. (2020). Enzyme mimic nanomaterials and their biomedical applications. Chembiochem 21 (17), 2408–2418. 10.1002/cbic.202000123 32227615

[B87] Sharanyakanth P. S. Radhakrishnan M. (2020). Synthesis of metal-organic frameworks (MOFs) and its application in food packaging: a critical review. Trends Food Sci. Technol. 104, 102–116. 10.1016/j.tifs.2020.08.004

[B88] Shi W. Li T. Chu N. Liu X. He M. Bui B. (2021). Nano-octahedral bimetallic Fe/Eu-MOF preparation and dual model sensing of serum alkaline phosphatase (ALP) based on its peroxidase-like property and fluorescence. Mater Sci. Eng. C Mater Biol. Appl. 129, 112404. 10.1016/j.msec.2021.112404 34579916

[B89] Shi X. Zhu G. Qiu S. Huang K. Yu J. Xu R. (2004). Zn2[(S)-O3PCH2NHC4H7CO2]2: a homochiral 3D zinc phosphonate with helical channels. Angew. Chem. Int. Ed. Engl. 43 (47), 6482–6485. 10.1002/anie.200460724 15578789

[B90] Singh S. (2019). Nanomaterials exhibiting enzyme-like properties (nanozymes): current advances and future perspectives. Front. Chem. 7, 46. 10.3389/fchem.2019.00046 30805331 PMC6370642

[B91] Stonesifer C. Corey S. Ghanekar S. Diamandis Z. Acosta S. A. Borlongan C. V. (2017). Stem cell therapy for abrogating stroke-induced neuroinflammation and relevant secondary cell death mechanisms. Prog. Neurobiol. 158, 94–131. 10.1016/j.pneurobio.2017.07.004 28743464 PMC5671910

[B92] Sugamata K. Yanagisawa D. Awano K. Iihama T. Minoura M. (2020). Structural analysis of interpenetrated methyl-modified MOF-5 and its gas-adsorption properties. Acta Crystallogr. C Struct. Chem. 76 (Pt 9), 845–849. 10.1107/s2053229620010177 32887853

[B93] Talebi M. Mohammadi Vadoud S. A. Haratian A. Talebi M. Farkhondeh T. Pourbagher-Shahri A. M. (2022). The interplay between oxidative stress and autophagy: focus on the development of neurological diseases. Behav. Brain Funct. 18 (1), 3. 10.1186/s12993-022-00187-3 35093121 PMC8799983

[B94] Tao W. Kong N. Ji X. Zhang Y. Sharma A. Ouyang J. (2019). Emerging two-dimensional monoelemental materials (Xenes) for biomedical applications. Chem. Soc. Rev. 48 (11), 2891–2912. 10.1039/c8cs00823j 31120049

[B95] Tuo Q. Z. Zhang S. T. Lei P. (2022). Mechanisms of neuronal cell death in ischemic stroke and their therapeutic implications. Med. Res. Rev. 42 (1), 259–305. 10.1002/med.21817 33957000

[B96] Villalba-Rodríguez A. M. Martínez-Zamudio L. Y. Martínez S. A. H. Rodríguez-Hernández J. A. Melchor-Martínez E. M. Flores-Contreras E. A. (2023). Nanomaterial constructs for catalytic applications in biomedicine: nanobiocatalysts and nanozymes. Top. Catal. 66 (9-12), 707–722. 10.1007/s11244-022-01766-4 36597435 PMC9798949

[B97] Wang D. Wu H. Phua S. Z. F. Yang G. Qi Lim W. Gu L. (2020). Self-assembled single-atom nanozyme for enhanced photodynamic therapy treatment of tumor. Nat. Commun. 11 (1), 357. 10.1038/s41467-019-14199-7 31953423 PMC6969186

[B98] Wang J. Wang Y. Xiaohalati X. Su Q. Liu J. Cai B. (2023). A bioinspired manganese-organic framework ameliorates ischemic stroke through its intrinsic nanozyme activity and upregulating endogenous antioxidant enzymes. Adv. Sci. (Weinh) 10 (20), e2206854. 10.1002/advs.202206854 37129343 PMC10369237

[B99] Wang L. Chen Y. (2020). Luminescence-Sensing Tb-MOF nanozyme for the detection and degradation of estrogen endocrine disruptors. ACS Appl. Mater Interfaces 12 (7), 8351–8358. 10.1021/acsami.9b22537 31965786

[B100] Wang L. Hu Z. Wu S. Pan J. Xu X. Niu X. (2020). A peroxidase-mimicking Zr-based MOF colorimetric sensing array to quantify and discriminate phosphorylated proteins. Anal. Chim. Acta 1121, 26–34. 10.1016/j.aca.2020.04.073 32493586

[B101] Wang L. Zhou H. Hu H. Wang Q. Chen X. (2022). Regulation mechanism of ssDNA aptamer in nanozymes and application of nanozyme-based aptasensors in food safety. Foods 11 (4), 544. 10.3390/foods11040544 35206017 PMC8871106

[B102] Wang M. Liu H. Ren J. Huang Y. Deng Y. Liu Y. (2023). Enzyme-assisted nucleic acid amplification in molecular diagnosis: a review. Biosens. (Basel) 13 (2), 160. 10.3390/bios13020160 PMC9953907 36831926

[B103] Wei H. Wang E. (2013). Nanomaterials with enzyme-like characteristics (nanozymes): next-generation artificial enzymes. Chem. Soc. Rev. 42 (14), 6060–6093. 10.1039/c3cs35486e 23740388

[B104] Wu J. Yu Y. Cheng Y. Cheng C. Zhang Y. Jiang B. (2021). Ligand-Dependent activity engineering of glutathione peroxidase-mimicking MIL-47(V) metal-organic framework nanozyme for therapy. Angew. Chem. Int. Ed. Engl. 60 (3), 1227–1234. 10.1002/anie.202010714 33022864

[B105] Wu Z. Adekoya D. Huang X. Kiefel M. J. Xie J. Xu W. (2020). Highly conductive two-dimensional metal-organic frameworks for resilient lithium storage with superb rate capability. ACS Nano 14 (9), 12016–12026. 10.1021/acsnano.0c05200 32833424

[B106] Wulff G. (2002). Enzyme-like catalysis by molecularly imprinted polymers. Chem. Rev. 102 (1), 1–28. 10.1021/cr980039a 11782127

[B107] Xia Y. Zhou J. Liu Y. Liu Y. Huang K. Yu H. (2022). Microplasma-assisted synthesis of a mixed-valence Ce-MOF with enhanced oxidase-like activity for colorimetric sensing of dopamine. Analyst 147 (23), 5355–5362. 10.1039/d2an01420c 36373378

[B108] Xiong X. Y. Liu L. Yang Q. W. (2016). Functions and mechanisms of microglia/macrophages in neuroinflammation and neurogenesis after stroke. Prog. Neurobiol. 142, 23–44. 10.1016/j.pneurobio.2016.05.001 27166859

[B109] Xiong Y. Chen S. Ye F. Su L. Zhang C. Shen S. (2015). Synthesis of a mixed valence state Ce-MOF as an oxidase mimetic for the colorimetric detection of biothiols. Chem. Commun. (Camb) 51 (22), 4635–4638. 10.1039/c4cc10346g 25690559

[B110] Xu D. Li C. Zi Y. Jiang D. Qu F. Zhao X. E. (2021). MOF@MnO(2)nanocomposites prepared usingin situmethod and recyclable cholesterol oxidase-inorganic hybrid nanoflowers for cholesterol determination. Nanotechnology 32 (31), 315502. 10.1088/1361-6528/abf692 33836512

[B111] Xu Q. Zhan G. Zhang Z. Yong T. Yang X. Gan L. (2021). Manganese porphyrin-based metal-organic framework for synergistic sonodynamic therapy and ferroptosis in hypoxic tumors. Theranostics 11 (4), 1937–1952. 10.7150/thno.45511 33408790 PMC7778611

[B112] Yan T. Zhang G. Yu K. Chai H. Tian M. Qu L. (2023). Smartphone light-driven zinc porphyrinic MOF nanosheets-based enzyme-free wearable photoelectrochemical sensor for continuous sweat vitamin C detection. Chem. Eng. J. 455, 140779. 10.1016/j.cej.2022.140779

[B113] Yang W. Zhang M. He J. Gong M. Sun J. Yang X. (2022). Central nervous system injury meets nanoceria: opportunities and challenges. Regen. Biomater. 9, rbac037. 10.1093/rb/rbac037 35784095 PMC9245649

[B114] Yu H. Han J. An S. Xie G. Chen S. (2018). Ce(III, IV)-MOF electrocatalyst as signal-amplifying tag for sensitive electrochemical aptasensing. Biosens. Bioelectron. 109, 63–69. 10.1016/j.bios.2018.03.005 29529509

[B115] Yu K. Chai H. Sun H. Xiang X. Zhao H. Tian M. (2024). A fluorescence analysis model for assessing the water stability of porphyrinic metal−organic frameworks. Sensors Actuators B Chem. 401, 135046. 10.1016/j.snb.2023.135046

[B116] Yu K. Li M. Chai H. Liu Q. Hai X. Tian M. (2023). MOF-818 nanozyme-based colorimetric and electrochemical dual-mode smartphone sensing platform for *in situ* detection of H2O2 and H2S released from living cells. Chem. Eng. J. 451, 138321. 10.1016/j.cej.2022.138321

[B117] Zhang K. Tu M. Gao W. Cai X. Song F. Chen Z. (2019). Hollow prussian blue nanozymes drive neuroprotection against ischemic stroke via attenuating oxidative stress, counteracting inflammation, and suppressing cell apoptosis. Nano Lett. 19 (5), 2812–2823. 10.1021/acs.nanolett.8b04729 30908916

[B118] Zhang L. Wang Z. Zhang Y. Cao F. Dong K. Ren J. (2018). Erythrocyte membrane cloaked metal-organic framework nanoparticle as biomimetic nanoreactor for starvation-activated colon cancer therapy. ACS Nano 12 (10), 10201–10211. 10.1021/acsnano.8b05200 30265804

[B119] Zhang P. Sun D. Cho A. Weon S. Lee S. Lee J. (2019). Modified carbon nitride nanozyme as bifunctional glucose oxidase-peroxidase for metal-free bioinspired cascade photocatalysis. Nat. Commun. 10 (1), 940. 10.1038/s41467-019-08731-y 30808912 PMC6391499

[B120] Zhang X. Li G. Wu D. Li X. Hu N. Chen J. (2019). Recent progress in the design fabrication of metal-organic frameworks-based nanozymes and their applications to sensing and cancer therapy. Biosens. Bioelectron. 137, 178–198. 10.1016/j.bios.2019.04.061 31100598

[B121] Zhang Y. Wang F. Liu C. Wang Z. Kang L. Huang Y. (2018). Nanozyme decorated metal-organic frameworks for enhanced photodynamic therapy. ACS Nano 12 (1), 651–661. 10.1021/acsnano.7b07746 29290107

[B122] Zhao J. Cai X. Gao W. Zhang L. Zou D. Zheng Y. (2018). Prussian blue nanozyme with multienzyme activity reduces colitis in mice. ACS Appl. Mater Interfaces 10 (31), 26108–26117. 10.1021/acsami.8b10345 30028115

[B123] Zhao S. Duan H. Yang Y. Yan X. Fan K. (2019). Fenozyme protects the integrity of the blood-brain barrier against experimental cerebral malaria. Nano Lett. 19 (12), 8887–8895. 10.1021/acs.nanolett.9b03774 31671939

[B124] Zhao Z. Pang J. Liu W. Lin T. Ye F. Zhao S. (2019). A bifunctional metal organic framework of type Fe(III)-BTC for cascade (enzymatic and enzyme-mimicking) colorimetric determination of glucose. Mikrochim. Acta 186 (5), 295. 10.1007/s00604-019-3416-7 31016397

[B125] Zhe Y. Zhang W. Gu C. Sun L. Dong F. Zhao Z. (2023). Bioinspired structure regulation of apyrase-like nanozyme with intracellular-generated H(2)O(2) for tumor catalytic therapy. ACS Appl. Mater Interfaces 15 (15), 19178–19189. 10.1021/acsami.3c00720 37023051

[B126] Zhong Y. Li Q. L. Lu M. Wang T. Yang H. He Q. (2020). A colorimetric sensing strategy based on enzyme@metal-organic framework and oxidase-like IrO(2)/MnO(2) nanocomposite for α-glucosidase inhibitor screening. Mikrochim. Acta 187 (12), 675. 10.1007/s00604-020-04660-6 33241461

[B127] Zhou Z. Xing X. Tian C. Wei W. Li D. Hu F. (2018). A multifunctional nanocage-based MOF with tri- and tetranuclear zinc cluster secondary building units. Sci. Rep. 8 (1), 3117. 10.1038/s41598-018-21382-1 29449641 PMC5814399

[B128] Zhu X. X. Guo D. F. Chen M. An X. Q. Wang B. Yu W. F. (2021). Application value and challenge of traditional Chinese medicine carried by ZIF-8 in the therapy of ischemic stroke. Ibrain 7 (4), 337–350. 10.1002/ibra.12007 37786560 PMC10529174

